# The Hospital Secretary

**Published:** 1909-01-16

**Authors:** 


### HOSPITAL ADMINISTRATION.

### CONSTRUCTION AND ECONOMICS.

### THE HOSPITAL SECRETARY.

HIS OFFICE, DUTIES, AND EMOLUMENTS.

The office of secretary to any undertaking whatever is one which the friends of almost every man anxious to secure a position and salary invariably consider him qualified to fill. This well-known fact illustrates how impossible it usually is to accept as reliable testimonials from the friends of candidates, and how essential it has become, if any regard is to be had to the efficient discharge of the duties of the secretarial office, that no one shall be regarded eligible for the secretariat unless he can produce satisfactory evidence of previous training and experience. One of the most amusing proofs of the truth of these contentions is provided by the well-accredited story of a vacant secretarial post connected with the Government some forty years ago. The post was an excellent one, the duties light and the salary handsome. The vacancy occurred in the days of patronage, and it is recorded that most members of the Cabinet and Opposition had candidates of their own. In the end a certain major was appointed to the post, and on inquiry it was found that the qualifications which brought him success consisted of varicose veins in the lower extremities. It was held that such a post was ideal for such a man, because he would be able to get rest for his limbs, and the appointment met with general approval. This instance is well authenticated, and we may add to it the fact that in forty years' experience, during which we have had to take part in the filling of many secretarial posts, our records show that it very often happens that the man who has the greatest number of friends is recommended for personal reasons, though they may have but little bearing on his fitness or capacity to discharge the duties of the office.

Of late years especially, so far as the more important hospitals are concerned, attempts have been made, as was the case at the Richmond Hospital a fortnight ago, to secure for these appointments men who have had training in the work, and who have displayed capacity and judgment which show them to be entitled to selection on their merits. The importance of the recognition of this principle by every hospital committee has been made very apparent by the enforcement of the adoption of the uniform system of accounts, with a balance sheet, by the great central funds in the metropolis, by the voluntary adoption of this system in the provinces, and by its use in the colonies by the governments there.

The hospital secretary to be efficient should be a man of excellent business training, good general knowledge, wide experience in dealing with men and women, proved administrative and financial abilities. He should possess a mastery of accounts, a familiarity with figures and the handling of statistics, and a multiplicity of other technical and general qualifications upon the possession of which by the secretary the economical and general efficiency of a hospital must greatly depend. When the secretary is really efficient he is necessarily a man with ambitions, who welcomes inquiries about executive and administrative matters from other hospitals and authorities seeking information at his hands. No really efficient secretary, however busy, will ever resent the receipt of such inquiries, nor fail to answer them to the best of his ability, providing they are genuine and practical. A secretary who arranges his office efficiently will provide for the ready dealing with technical correspondence of this kind, and so prove his capacity and win for himself recognition in the hospital world and at the hands of those who have it in their power to promote the progress and reputation of the best workers in the hospital field.

Until the King's Fund actively supported the uniform system of accounts, and strenuously enforced the accurate keeping of the books and the rendering of statistical information upon intelligent lines, there were hospitals in London where certain officials dared to fill in the forms of the Hospital Sunday Fund Council, for example, as if they used the uniform system, although the books might be kept upon the single-entry or some other system, or upon no system at all. Any secretary guilty of such practices is self-condemned as hopelessly inefficient. Indeed our view is that hospital committees should make it their business to know, through the active supervision of a Finance and Economic Committee, how the books are kept, and also to know what information of a statistical and other kind is sought from outside at the hands of their secretary, and to provide that this work is well and efficiently done. Indeed the best-managed hospitals now have some such system in force. Nevertheless, there are one or two instances in the secretarial world still of men who are too lazy or two unintelligent to supply adequate statistical information, and it is within our knowledge that their hospitals have lost through such inefficiency large sums of money within the last fifteen years.

The action of the King's, the Sunday and Saturday Funds in agreeing to one common statistical form, in which the material financial facts relating to each year's work are to be returned, is a welcome intimation on the part of those in authority that there is every wish at all times to reduce and not to add to the work of the secretary of a great modern hospital. The system adopted by some of the principal and most efficient hospital secretaries, who have laid themselves out to fill the higher offices in their department by selecting men of education and ability, and to provide for their adequate training in the duties of a hospital secretary, is to be warmly commended. Every hospital secretary who adopts this plan displays to a marked extent his special capacity for the work he has undertaken, for his action is a recognition that, after all, the hospital secretary has the high privilege of rendering important public service in these days. For this reason we have held for many years, and have always maintained, that every efficient hospital committee should take care to see that the hospital secretary and superintendent is adequately remunerated for his services, and that a proper provision is made to secure him a pension on a contributory basis or otherwise when the age of retirement arrives. There are very few groups of men attached to public institutions who, on the whole, render more valuable services to the community than hospital secretaries.

For these reasons we are glad to be able to report that the Committee of University College Hospital, which institution has never attained to the financial strength and position which it ought long ago to have occupied amongst metropolitan hospitals, have had the wisdom to appoint to the existing vacancy Mr. J. G. Buckle, B.A. Cantab., who passed third into the Colonial Service and held the post of Assistant Colonial Secretary at Hong Kong for some years. On leaving that service he became assistant secretary at King's College, and subsequently assistant secretary to the Seamen's Hospital at Greenwich, where Mr. Buckle gained a thorough insight into the modern system of hospital administration and most things which tend to secure the efficient working of a great hospital. We heartily congratulate the Committee of University College Hospital and Mr. J. G. Buckle upon this appointment, and we have every confidence that it will be justified in the near future by the results which are likely to follow from this important step. We hope the days have gone by when any hospital committee will disregard previous training and adequate technical and general knowledge as paramount qualifications for the post of secretary.

